# Anæsthesia

**Published:** 1870-06

**Authors:** G. Q. Colton

**Affiliations:** New York


### ANÆSTHESIA.

BY G. Q. COLTON.

I have perused, with much interest, the article "Anæsthesia in Surgery—Who Discovered it?" published in the April number of the Dental Register. I agree entirely with the writer that the honor of that discovery belongs alone to Dr. Horace Wells; but the article contains some errors, and omits some important facts connected with the subject, which I will briefly endeavor to correct.

At the close of the exhibition of the gas which I gave in Hartford, on the 10th of December, 1844, at which a young man (I think it was not Col. Cooley) bruised himself against some benches, but experienced no pain. Dr. Wells asked me why a tooth could not be drawn with the gas without pain. I replied that the thought had never occurred to me. I then remembered and stated that at an exhibition which I gave at Bridgeport, a few evenings previous, a young man broke one of the bones of his hand, but said he felt no pain till the effects of the gas had passed off. Dr. Wells at once decided to try the experiment on himself, and arranged with me to bring a bag of the gas to his office the next day. He had a molar tooth, slightly decayed, which he was willing to part with. Accordingly the next day I took a bag of the gas to Dr. Wells' office. Dr. Riggs was called in, when I administered the gas to Dr. Wells, and Dr. Riggs drew his tooth. The statement that "Dr. Wells took the pipe of the gas-bag in his mouth, and it was kept there till his hands fell lifeless and his head drooped," is slightly incorrect, as Dr. Wells did not touch the "pipe" with his hands, neither did his hands fall lifeless or his head droop. I used but a small bag, such as would ordinarily produce exhilaration. On recovering, Dr. Wells exclaimed: "I did not feel it so much as the prick of a pin!" He would not have felt even this if his head had "drooped."

I think the statement about the consultation lasting till midnight, between Dr. Wells and Dr. Riggs, must be incorrect, for I saw nothing of Dr. Riggs that evening, and Dr. Wells walked home with his wife (according to her testimony) after the exhibition.

Dr. Wells was delighted with his discovery, and wished me to instruct him how to make the gas, and to furnish him with the apparatus. I gave him the instructions, but had no time to get up the apparatus.

Dr. Wells died in this city January 24th, 1848. Nearly two years of the time from the period of his discovery till his death, he spent in Europe in search of health. During nearly all these four years he was experimenting successfully with the nitrous oxide, using it constantly during his practice at Hartford. But with all his efforts, he failed to convince the medical or Dental professions of the great value of the gas as an anæsthetic. With his death, all use of the gas ceased, and even Dr. Riggs dropped it. It was condemned, passed out of the public mind, and was forgotten. In June, 1863, remembering the experiment with Wells, I determined to revive, and if possible demonstrate the value of this gas as an anæsthetic. I found it hard and up-hill work the first year, but the tide at length turned in its favor. While I claim no special merit for this revival, yet I think it should be remembered that the gas had passed out of public notice and laid dead for seventeen years, and *possibly* might never have been thought of again but for my efforts. So far as the public is concerned, the revival produced all the benefits of a new discovery. Yet I claim none of the honor of the discovery. This belongs to Dr. Wells.

I had the pleasure of subscribing five hundred dollars towards securing the publication of the work on "Modern Anæsthesia," by the Hon. Truman Smith, and I believe it was the extensive circulation of this book in this country and in Europe which has carried the day in favor of Dr. Wells, as the original discoverer of anæsthesia.

The rather gasy advertisement which is copied from the *Hartford Times*, is, I presume, about what I should have written twenty-six years ago. But its puffy nature will be excused when it is remembered that it attracted the attendance of Dr. Wells, and was the occasion of the most important and valuable discovery of the present century.

I sincerely trust that the movement in favor of erecting a monument to the memory of Dr. Wells will result in triumphant consummation.

New York, May 20th, 1870.

---

Perhaps the greatest triumph of the art of medicine of the present age, is the use of remedies hypodermically. Its chief merits are promptitude of action, thus gaining much *eclat* to the physician, by his absolute command over pain, which is usually borne badly, even by the most stoical. Armed with his syringe, the physician becomes a conqueror worthy of the name. It matters not whether the stomach will bear the presence of the remedy or not, this is a short-cut to the disease or pain, allowing the oft-sinned-against stomach time to rest. Another well-established fact is that the remedy does not lose its power through force of habit, requiring no increase of dose.

